# Mechanisms involved in the anti-tumor effects of Toosendanin in glioma cells

**DOI:** 10.1186/s12935-021-02186-2

**Published:** 2021-09-16

**Authors:** Chaochao Zhang, Haijun Gao, Ziqiang Liu, Jiacheng Lai, Zhixin Zhan, Yong Chen, Haiyan Huang

**Affiliations:** Department of Neurosurgery, The First Hospital of Jilin University, Changchun, 130021 China

**Keywords:** Toosendanin, Glioma, Apoptosis, Proliferation, PI3K, Akt, mTOR pathway

## Abstract

**Background:**

Toosendanin (TSN) is a triterpenoid compound mainly used as an ascaris repellant. Recent studies have shown that it possesses antitumor effects in many types of tumor cells. However, the effects of TSN on glioma cells have rarely been reported.

**Methods:**

Different assays were performed to investigate the effects of TSN on the different glioma cell lines including U87MG and LN18. The assays included colony formation, wound healing, and transwell assays. Furthermore, Hoechst 33342 staining, flow cytometry, and western blotting analysis were performed to investigate the apoptotic activities of TSN. Finally, the results were confirmed using a xenograft tumor model that comprised of nude mice.

**Results:**

In vitro, the CCK-8 and colony formation assays showed that TSN effectively inhibited glioma cell proliferation. Moreover, the inhibitory effects on glioma cell migration and invasion were demonstrated through the wound healing and transwell assays, respectively. Hoechst 33342 staining, flow cytometry, and western blotting assays demonstrated the significant effect of TSN in the apoptosis induction of glioma cells. Furthermore, the anti-glioma effect of TSN was exerted through the inhibition of the PI3K/Akt/mTOR signaling pathways as demonstrated by western blotting analysis. In addition, the effects of TSN on glioma cell viability, apoptosis, cell cycle arrest, migration, and invasion were reversed by 740Y-P, a PI3K activator. Finally, the mouse xenograft model confirmed the suppressive effect of TSN on tumor growth in vivo.

**Conclusion:**

Our results suggest that TSN is a promising chemotherapeutic drug for patients with glioma.

## Introduction

Gliomas are tumors originating from brain glial cells such as astrocytes, oligodendrocytes, and ependymal cells. Among its different types, glioblastomas (GBM) are the most common intracranial tumor with the highest degree of malignancy, accounting for approximately half of the incidence, i.e., 3–4 per 100,000 [1]. The classic Stupp protocol is the most common and effective treatment for GBM. This protocol includes total or subtotal resection within the scope of safety and postoperative conventional radiotherapy plus adjuvant chemotherapy using temozolomide [2, 3]. In addition, other treatments include tumor treating fields, targeted medication, immunotherapy, and traditional Chinese medicine [4, 5]. However, the high degree of malignancy, rapid growth, and high recurrence rate of GBM has resulted in an extremely low survival rate despite completion of active treatment. A study found < 13% 5-year survival rate and < 20.5 months survival time in patients with GBM [6]. Therefore, relevant preclinical studies are warranted to determine an effective and safe treatment for GBM.

Toosendanin (TSN) is a tetracyclic triterpenoid derived from the Chuan neem seed or blast. It is mainly used as an ascaris repellent; additionally, it has biological activities including anti-botulism, neuromuscular junction inhibition, and central nervous and respiratory system effects [7]. A large number of studies have shown the efficacy of natural products in cancer treatment, particularly, in cancer drug research [8–10]. Among them, TSN has recently been shown to possess anti-tumor activity in many tumor cells including gastric, lung, pancreatic, colorectal, liver, and breast cancer cells [11–17]. However, studies regarding the major pharmacological and molecular mechanisms of TSN on glioma cells remain lacking.

Therefore, this study aimed to explore the anti-tumor effects of TSN on glioma cell lines (U87MG and LN18) in vivo and in vitro and potentially elucidate the underlying mechanisms.

## Methods

### Reagents

TSN (purity of approximately 99%) was purchased from Chroma Biotechnology Company (Chengdu, China). TSN was dissolved in dimethyl sulfoxide (DMSO), which was diluted with intact cell culture medium to achieve the concentration required for the experiment. The final concentration of DMSO was < 0.1% to avoid side effects.

Dulbecco’s modified Eagle’s medium (DMEM) and fetal bovine serum (FBS) were purchased from Thermo Fisher (Waltham, MA, USA). Penicillin/streptomycin (100×) and phosphate-buffered saline (PBS) were purchased from Shanghai Basal Media Technologies Co., Ltd. (Shanghai, China). Cell counting Kit-8 (CCK-8), BCA Protein Assay Kit, Annexin V-FITC Apoptosis Detection Kit, Hoechst Staining Kit, Cell Cycle and Apoptosis Analysis Kit, electrochemiluminescence (ECL) kit, and Tris-buffered saline with Tween-20 (TBS-T) were purchased from Beyotime Biotechnology Company (Shanghai, China). 740Y-P was purchased from TargetMol (Shanghai, China). Primary antibodies against cleaved caspase-3, Bax, cyclin D1, p21, matrix metalloproteinase-2 (MMP2), phosphoinositol 3-kinase (PI3K), protein kinase B (Akt), and phosphorylated Akt (p-Akt) were purchased from Cell Signaling Technology (Danvers, MA, USA). Primary antibodies against PARP-1, phosphorylated PI3K (p-PI3K), mammalian target of rapamycin (mTOR), phosphorylated mTOR (p-mTOR), matrix metalloproteinase-9 (MMP9), Bcl-2, cleaved caspase-9, p27, CDK-4, and CDK-6 were purchased from Abcam (Cambridge, MA, UK). Secondary antibodies (goat anti-rabbit IgG and goat anti-mouse IgG) were obtained from Cell Signaling Technology (Beverly, MA, USA).

### Cell lines and cell culture

Human GBM cell lines U87MG, LN18, U251, LN229, and SVG p12were purchased from American Type Culture Collection (ATCC) and preserved in our laboratory at the Translational Medicine Institute of the First Hospital of Jilin University. All cell lines were cultured and maintained in DMEM supplemented with 10% FBS and antibiotics, including 1% streptomycin (100 μg/mL) and 1% penicillin (100 U/mL) in a humidified atmosphere of 5% CO_2_ at 37 °C.

### Cell viability analysis

The cell suspensions of U87MG, LN18, U251, LN229, and SVG p12cells were seeded into 96-well plates at a density of 7 × 10^3^ cells per well. The cells were incubated overnight, which allowed adherence to the wall at 37 °C. The original cell culture medium was replaced with fresh medium containing different concentrations of TSN. After 24, 48, and 72 h of drug treatment, 10 µl CCK-8 solution was added to each well (avoiding air bubbles). Incubation was extended for 1.5 h, after which the optical density (OD) value was measured and recorded at 460 nm.

### Colony formation assays

U87MG and LN18 glioma cells were seeded into 6-well plates at a density of 800 cells per well, which were cultured overnight in a CO_2_ humidified incubator. The culture medium containing the desired drug concentrations was added to each well. After 24 h, the medium was removed, and fresh medium was added to each well every 3 days for approximately 2 weeks. When colony formation was visible, they were fixed with ice-cold paraformaldehyde for 20 min and then stained with crystal violet for 20 min. Colonies with > 50 cells were observed under a microscope.

### Wound healing assays

U87MG and LN18 cells were inoculated into 6-well plates and cultured until the cell density reached approximately 80–90%. A sterile 200 μL was used to vertically scratch the plate surface and achieve similar width of cell wounds. Fresh serum-free medium containing different concentrations of TSN was added after discarding the original culture medium. The scratch area of each group was recorded separately by an inverted microscope at 0 and 48 h. The measurement of the wound healing area was performed using Image J software (version 1.52i, NIH, Bethesda, USA).

### Cell invasion assays

The surface of the transwell upper chamber was evenly coated with the matrix and precooled serum-free solution at a ratio of 1:9 for 6 h. Then, 70 μL of serum-free medium was added to hydrate the basement membrane for 30 min. U87MG and LN18 cells treated with the indicated concentration of TSN with a density of 1 × 10^4^ cells per well were seeded in the upper chamber using 180 µL of serum-free medium; then, 700 µL medium containing 10% FBS was added to the lower chamber. After conventional incubation for 16 h, the residual matrix glue and cells in the upper chamber were lightly removed with cotton swabs; the chamber was then fixed with methanol for 15 min. The chamber was removed, the membrane was dried in air, and then stained with 1% crystal violet for 30 min. Finally, the invasive cells were observed, photographed, and quantified in three random fields under an upright microscope (Olympus IX53/DP80, Tokyo, Japan).

### Cell cycle analysis

U87MG and LN18 cells treated with TSN for 48 h were collected and fixed with 70% pre-cooled ethanol at − 20 °C for at least 24 h. Cells stained with propidium iodide (PI) and RNase for 20 min were detected by flow cytometry (Becton Dickinson, San Diego, CA, USA).

### Hoechst apoptosis staining

U87MG and LN18 cells were seeded in 6-well plates at a density of 5 × 10^4^ cells per well. After the cells were adherent to the wall, different concentrations (0, 50, 100, and 200 μM) of TSN were added to each well. After 24 h, the cells were stained with Hoechst 33342 (1 ug/mL) for 15 min under light-proof conditions and observed under a fluorescence microscope at 200× magnification; then, the number of apoptotic cells in each field was counted and analyzed.

### Annexin V-FITC/PI double staining assay

The Annexin V-FITC/PI cell apoptosis detection kit was used to observe the effect of TSN on the apoptosis of human glioma cells. U87MG and LN18 cells were seeded in a 6-well plate at a concentration of 4 × 10^5^ cells/mL. After treatment with the corresponding TSN for 48 h, the cells with 1 × binding buffer (100 μL) were incubated with PI (5 μL) and Annexin V-FITC (5 μL) at room temperature for 20 min. Subsequently, 1 × binding buffer (400 μL) was added, and the apoptosis level of glioma cells was detected using flow cytometry. The apoptosis rate was analyzed using FlowJo software (version 10.3, TreeStar, USA).

### Western blotting analysis

After glioma cells were treated with different concentrations of TSN for 48 h, the cells were lysed to extract the total protein. The protein concentration was determined using a BCA kit. A total of 30 mg of protein was loaded per lane onto an SDS-PAGE gel (8–10%). The protein in the gel was transferred to the PVDF membrane using a wet transblotting system (Bio-Rad, USA). The membrane was blocked in 5% skimmed milk powder for at least 1 h. It was left to incubate overnight with the appropriate primary antibody at 4 °C on a shaker. Subsequently, the membranes were incubated with the appropriate secondary antibody at room temperature for 2 h. The exposure of chromogenic protein bands with ECL luminescent solution was photographed and analyzed using a gel imaging system.

### Xenograft tumor model

5-week-old, male, specific pathogen-free (SPF)-level, athymic, immunocompromised (BALB/c nude) mice were obtained from Charles River Laboratories (Beijing, China). Nude mice were reared carefully in the SPF animal laboratory of The First Hospital of Jilin University. Under sterile conditions, a 100-μL suspension of U87MG cells (1 × 10^7^ cells/mouse) was inoculated subcutaneously into the right armpit of nude mice. When the tumor size reached approximately 180 mm^3^, the mice were randomly divided into two groups with five mice in each group. The mice from the experimental and control groups were intraperitoneally injected with 1.0 mg/kg TSN and the same volume of saline, respectively, every day for consecutive 21 days.

The weight of each mouse was measured every 3 days, and the longest (L) and shortest diameters (S) of the tumors were recorded. The tumor volume was calculated according to the standard formula: tumor volume = 0.5 × L × S^2^. The following day, the nude mice were sacrificed by the cervical dislocation method. The tumor tissue was harvested and photographed, and the weight of the tumors was measured. The tumor was divided into two sections: one was used for immunochemistry, and the other was preserved using liquid nitrogen for standby use. Additionally, the heart, liver, spleen, lung, kidney, and jejunum of the mice were removed for HE staining.

### Hematoxylin and eosin (H&E) and immunohistochemistry

The organs were fixed with 4% formaldehyde for 24 h, embedded in paraffin, cut into 4 pieces-μm thick sections, and dehydrated. The sections were then stained with H&E. In addition, some tumor slices were immunostained with the, Ki-67 (1:500) antibody, and p-Akt (1:100) antibody. Digital images were observed and captured under a standard optical microscope.

### Statistical analysis

All experimental results were obtained from at least three independent experiments using GraphPad Prism 8.3 (GraphPad Software, Inc., La Jolla, CA, USA). Data are presented as the means ± standard deviation (SD). Student’s t-test was used to analyze data between groups, while one-way ANOVA was used to analyze the differences between multiple groups. Statistical significance was set at p < 0.05.

## Results

### TSN inhibited glioma cells proliferation

**Fig. 1:**
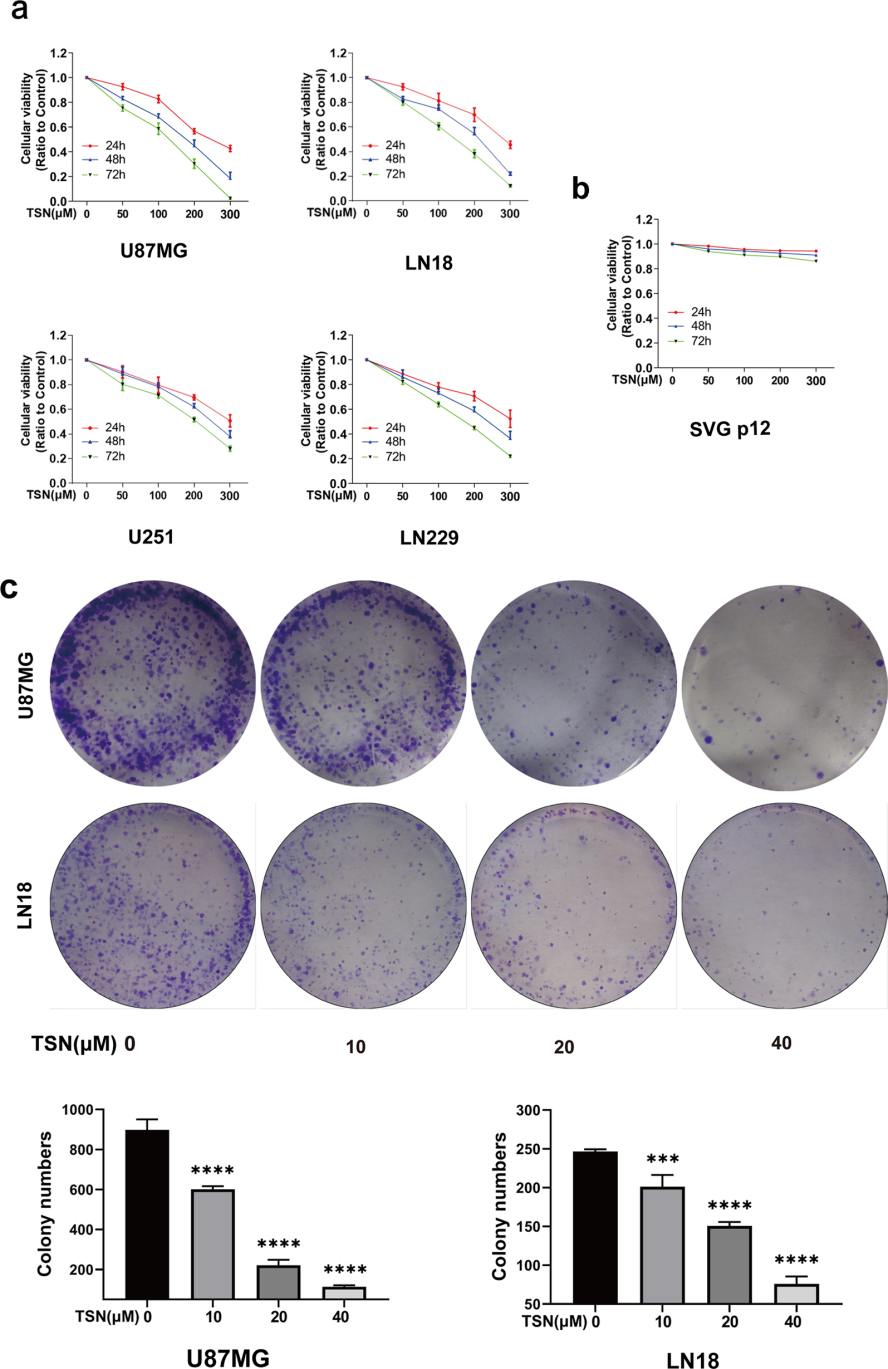
Toosendanin (TSN) inhibited the proliferation of glioma cells. **a** The chemical structure of TSN. **b**, **c** The viability of U87MG, LN18, U-251MG, and LN229 glioma cells and normal human astrocytes (SVG p12 cell) treated with different concentrations of TSN (0, 50, 100, 200, and 300 μM) for 24, 48, and 72 h was detected using CCK-8 assay. **d**, **e** Colony formation assay was performed to detect the colony numbers of U87MG and LN18 cells. ***P < 0.001, ****P < 0.0001 vs. TSN at 0 μmol/L. All experiments were implemented in triplicate

Glioma cells were treated with different concentrations of TSN (0, 50, 100, 200, and 300 μM) to evaluate the effect of TSN. The CCK-8 assay results showed a dose- and time-dependent decrease on the viability of glioma cells when TSN concentration increased (Fig. 1a). The half inhibitory concentration (IC50) of TSN for 48 h on U87MG, LN18, LN229, and U251 cells were 114.5, 172.6, 217.8, and 265.6 µM, respectively. The inhibitory effect of viability of U87MG and LN18 was evidently better compared to the other two cell lines. Additionally, the cytotoxicity of TSN to normal human astrocytes (SVG p12) was evaluated. The CCK-8 assay showed that TSN had no significant effect and had limited toxicity to normal human astrocytes (Fig. 1b). In addition, a colony formation assay was performed to evaluate the effect of TSN on the proliferation and long-term toxicity of glioma cells (Fig. 1c). The results showed a significant, dose-dependent increase in the number, size, and colony formation percentage in the U87MG and LN18 cells treated with low-dose TSN compared to the control.

### TSN inhibited glioma cells migration and invasion

**Fig. 2:**
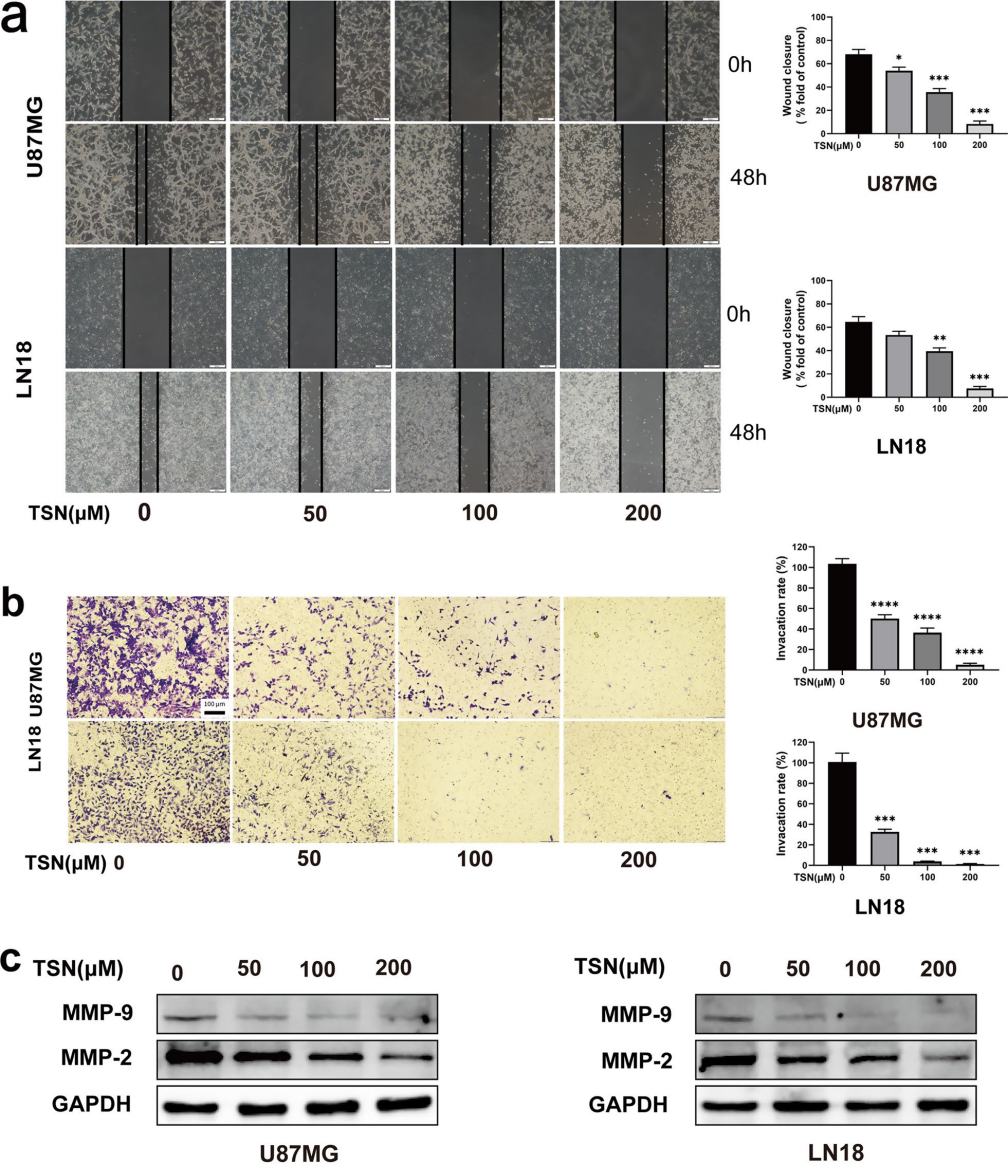
TSN inhibited the migration and invasion of glioma cells. **a** The wound healing experiment was performed to evaluate the migration capability of U87MG and LN18 cells under various concentrations of TSN (0, 50, 100, and 200 μM) at 0 h and 48 h. **b** Furthermore, transwell assay was performed using the same concentrations of TSN to evaluate the effect of TSN on the invasion of glioma cells. **c** Finally, western blotting assay was performed to detect the expression of migration- and invasion-related proteins, MMP-2 and MMP-9. (*P < 0.05, **P < 0.01, ***P < 0.001, ****P < 0.0001 vs. TSN at 0 μmol/L)

We performed wound healing and transwell assays to evaluate the effect of TSN on the migration and invasion of glioma cells. TSN significantly inhibited the wound healing (Fig. 2a) and invasion ability (Fig. 2b) of U87MG and LN18 cells in a dose-dependent manner. In the control group of the U87MG cell line, the wound healing rate was 58.5%; however, after treatment with 50, 100, and 200 µM after 48 h, the wound healing rate was 47.0%, 36.4%, and 8.2%, respectively. The wound healing rate of the LN18 cells control group was approximately 64.8%; treatment using 50, 100, and 200 µM TSN after 48 h led to wound healing rates of 51.1%, 36.4%, and 5.6%, respectively.

Transwell assays showed similar results for invasion. Additionally, we detected the expression levels of key proteins, MMPs, which regulate cell migration and invasion [18]. We found that TSN significantly inhibited the expression of MMP-2 and MMP-9 in a dose-dependent manner (Fig. 2c). These results show the anti-migration and anti-invasion effect of TSN on glioma cells.

### TSN induced apoptosis in glioma cells

**Fig. 3:**
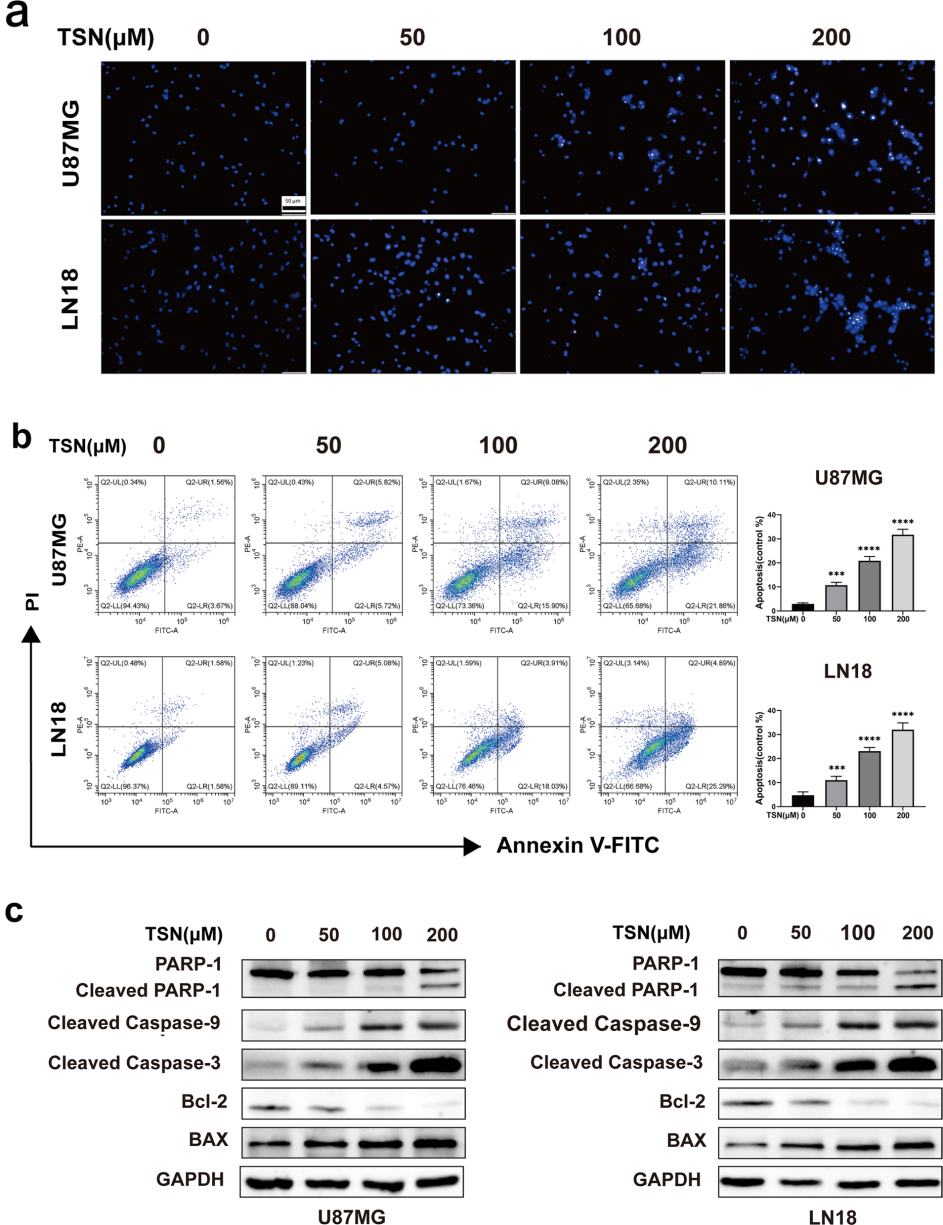
TSN induced apoptosis in glioma cells. **a** Hoechst 33342 staining was performed to detect apoptosis and the nucleic changes in U87MG and LN18 cells which were observed under a fluorescence microscope after 24 h treatment with TSN. **b** Flow cytometry was performed to further evaluate the effect of TSN on the apoptosis of glioma cells, which showed significant increases in the number of apoptotic cells. **c** Next, certain apoptosis-related proteins were detected by western blotting. (***P < 0.001, ****P < 0.0001 vs. TSN at 0 μmol/L)

Apoptosis is programmed cell death; escaping this event leads to unlimited tumor cell growth [19]. Hoechst 33342 staining showed nuclear pyknosis, dense staining, and apoptotic bodies in U87MG and LN18 cells after 24 h of TSN treatment (Fig. 3a). Flow cytometry results showed a dose-dependent increase in the number of early and late apoptotic cells after TSN treatment (Fig. 3b). Next, we detected apoptosis-related proteins by western blotting. Compared with the control group, TSN significantly increased the expression of Bax, cleaved Caspase-3/9, and cleaved PARP-1 proteins, but decreased the expression of Bcl-2 protein in a dose-dependent manner (Fig. 3c).

### TSN induced G0/G1 phase cell cycle arrest in glioma cells

**Fig. 4:**
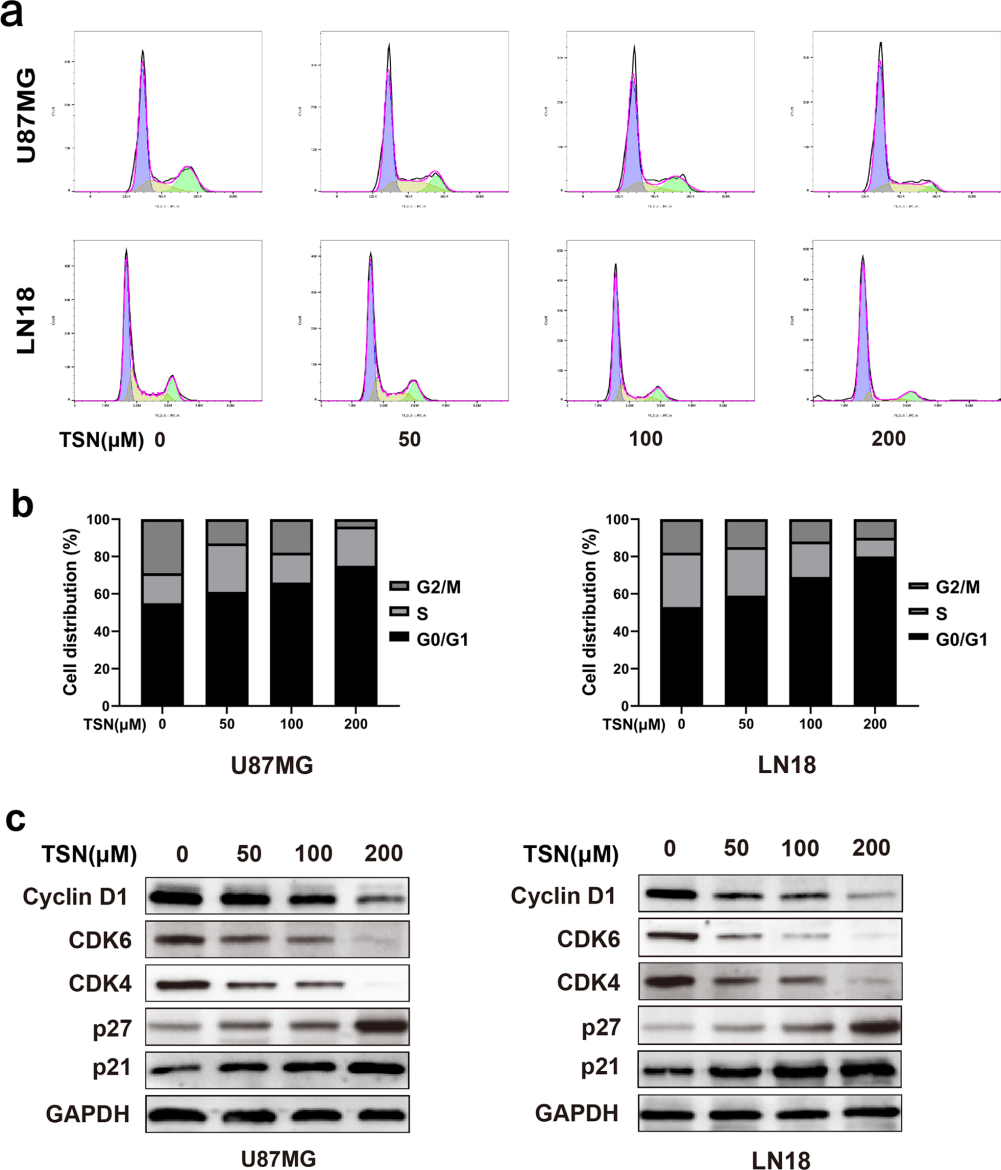
TSN induced G0/G1 phase cell cycle arrest in glioma cells. **a** The cell cycle of U87MG and LN18 cells treated with different concentrations for 48 h was detected by flow cytometry, which showed significant increases in the proportion of cells in the G0/G1 phase. **b** The expression levels of some cell cycle related-proteins cyclin D1, CDK4/6, p21, and p27 were detected using western blotting

Flow cytometry can detect DNA content in cells and analyze the mitotic cycle distribution of the cells. The results showed changes in the proportion of cells in the G0/G1 phase in glioma cells treated with different concentrations of TSN after 48 h. Furthermore, increasing the concentration of TSN leads to a significant increase in the proportion of glioma cells arrested in the G0/G1 phase (Fig. 4a). To further explore the mechanism, we detected the expression levels of certain cell cycle-related proteins using western blotting. This revealed the downregulation of cyclin D1 and CDK4/6 proteins and the upregulation of p21 an p27 proteins. (Fig. 4b). These data demonstrate that TSN induces G0/G1 arrest by modulating cell cycle-related protein levels.

### TSN inhibited the PI3K/Akt/mTOR signaling pathway in glioma cells

**Fig. 5:**
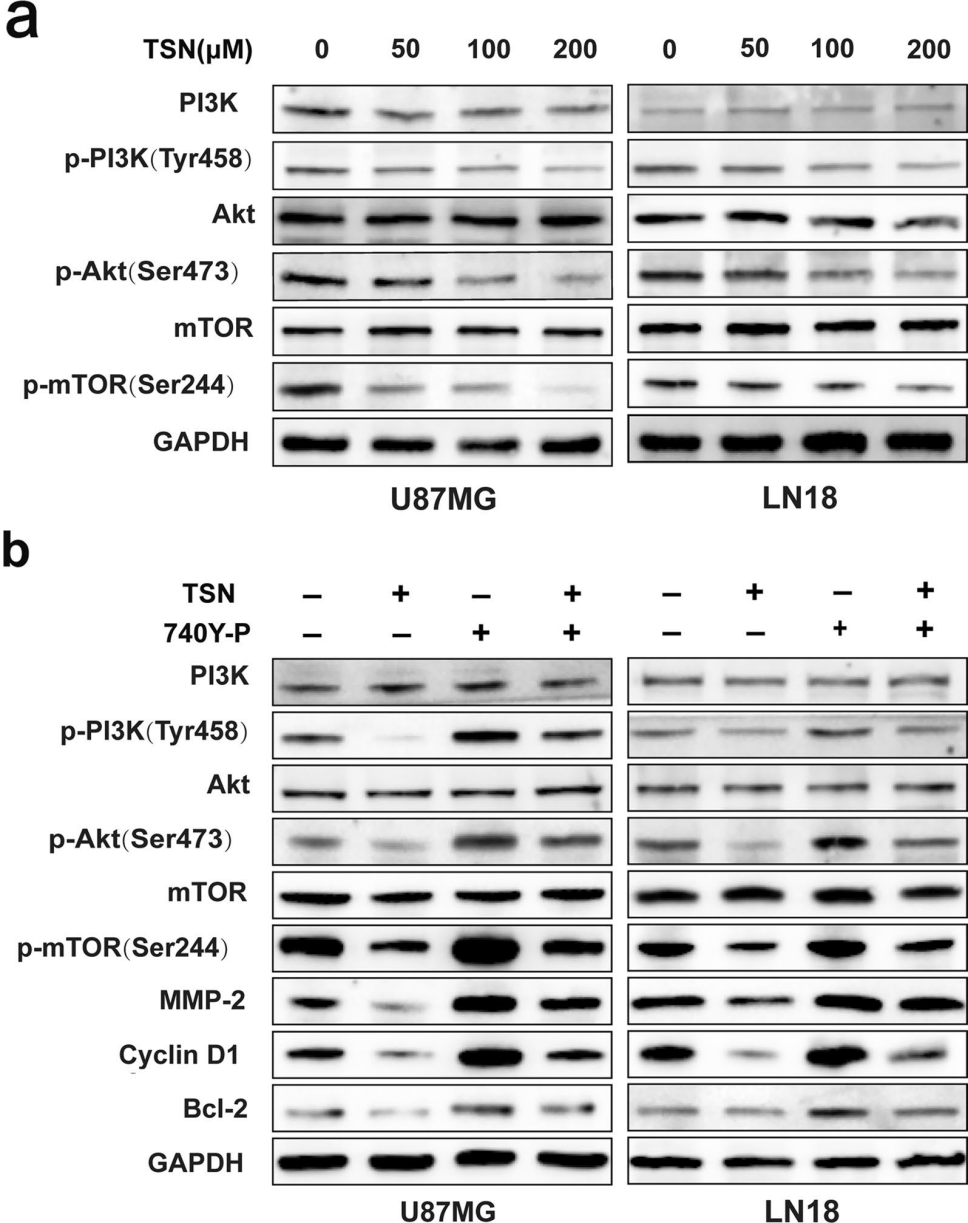
TSN induced cell viability, migration, invasion, and apoptosis by inhibiting the PI3K/Akt/mTOR signaling pathway. **a** Western blotting assay was performed to measure the expression levels of phosphorylated and non-phosphorylated PI3K, Akt, and mTOR proteins in U87MG and LN18 cells. **b** Then, the same cells were treated with 15 μM 740Y-P alone or 100 μM TSN alone or combined with 15 μM 740Y-P for 48 h; the same assay was performed to detect the protein levels of p-PI3K, PI3K, p-Akt, Akt, p-mTOR, and mTOR, MMP-2, cyclin D1, and Bcl-2

The above results demonstrated that TSN can significantly regulate the expression levels of tumor migration- and invasion-related, cell-cycle-related, and apoptosis-related proteins. To further explore the molecular mechanism involved, western blotting analysis was performed. The results showed that TSN significantly inhibited the phosphorylation levels of PI3K, Akt, and mTOR proteins in a dose-dependent manner. However, no significant effect was observed regarding the total protein levels of PI3K, Akt, and mTOR (Fig. 5a). After phosphorylation of PI3K, Akt, and mTOR in sequence, some downstream signaling molecules regulating cell proliferation and metastasis, apoptosis, and cell cycle were activated (Figs. 2c, 3c, and 4b). However, we found that 749Y-P, a PI3K activator, reversed the inhibitory effect of TSN on PI3K/Akt/mTOR (Fig. 5b).

### TSN inhibited glioma cell proliferation in vivo

**Fig. 6:**
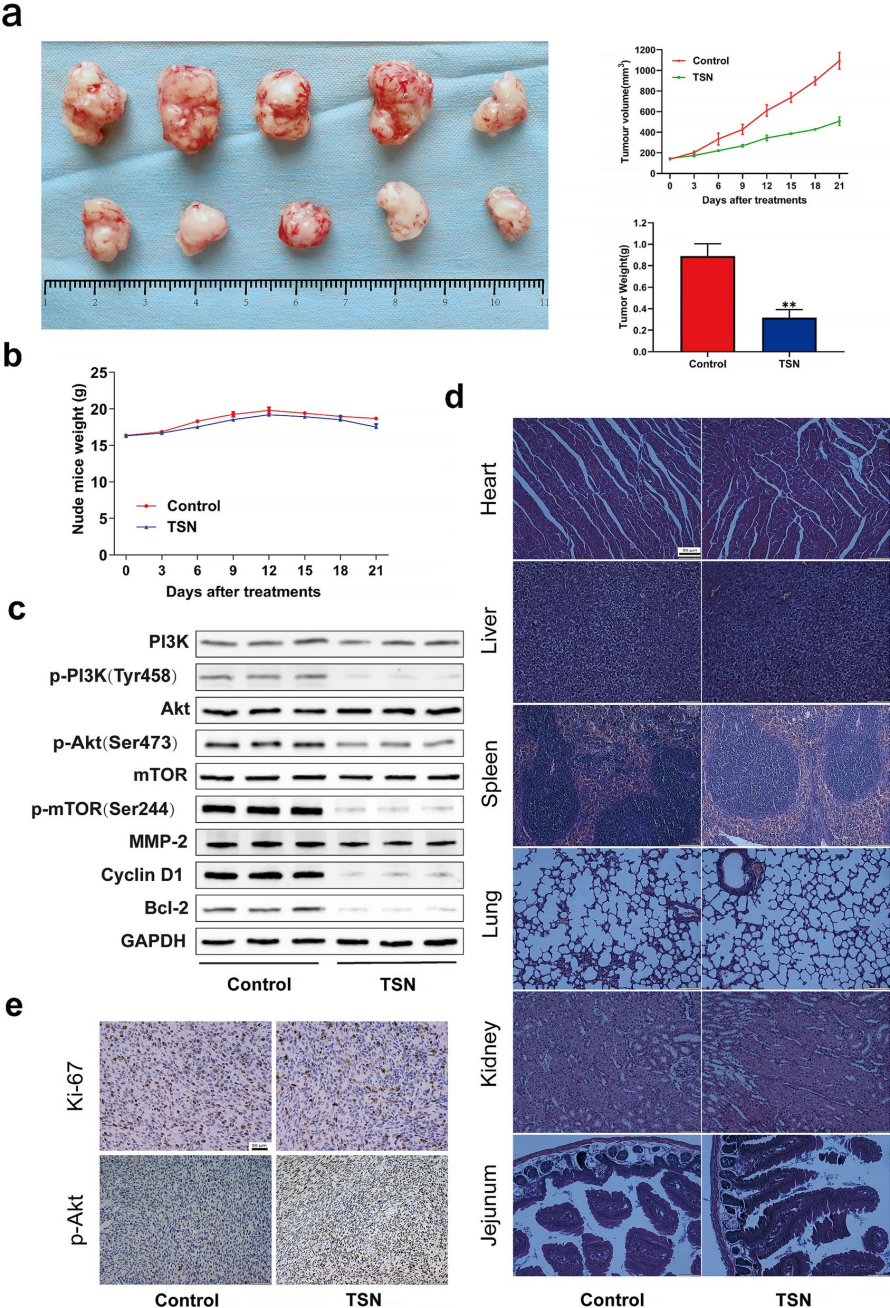
TSN inhibited tumor growth in the xenograft model mice. **a** The tumors of control mice and TSN-treated mice were extracted and photographed. Inhibitions in the average volume and weight of glioma xenografts were observed after 21 days of TSN treatment. **b** Body weight changes of mice during 21 days of exposure. **c** Western blotting of tumor tissue lysates. **d** H&E staining of main organs in the control and TSN-treated mice. Immunohistochemical staining of p-Akt and Ki-67 (**e**). **P < 0.01, significantly different compared with the vehicle control group

All the nude mice grew well throughout the experimental process; none of the mice demonstrated evidence of infection, weight loss, adverse effects, or death. Gradual increase in the tumor volume of the nude mice was observed as the experiment progressed. The tumors in the control group were evidently larger compared to the TSN treatment group; particularly, an evident reduction in the average tumor volume and weight was observed in the TSN group (Fig. 6a). However, no significant difference was observed regarding the body weight between the two groups (Fig. 6b).

The western blotting assays of tumor tissue lysates showed that TSN inhibited the PI3K/Akt/mTOR signaling pathway and the expression of MMP-2, cyclin D1, and Bcl-2 proteins (Fig. 6c). Additionally, no evident pathological changes were observed in the main organs of the two groups, indicating the low toxicity of TSN in normal tissues (Fig. 6d). Finally, immunohistochemical analysis showed the inhibition of the Ki-67 and p-Akt expression in the TSN-treated group compared to the control (Fig. 6e). Collectively, these results demonstrated TSN inhibited glioma tumor progression by targeting PI3K/AkT/mTOR signaling pathways in vivo.

## Discussion

**Fig. 7:**
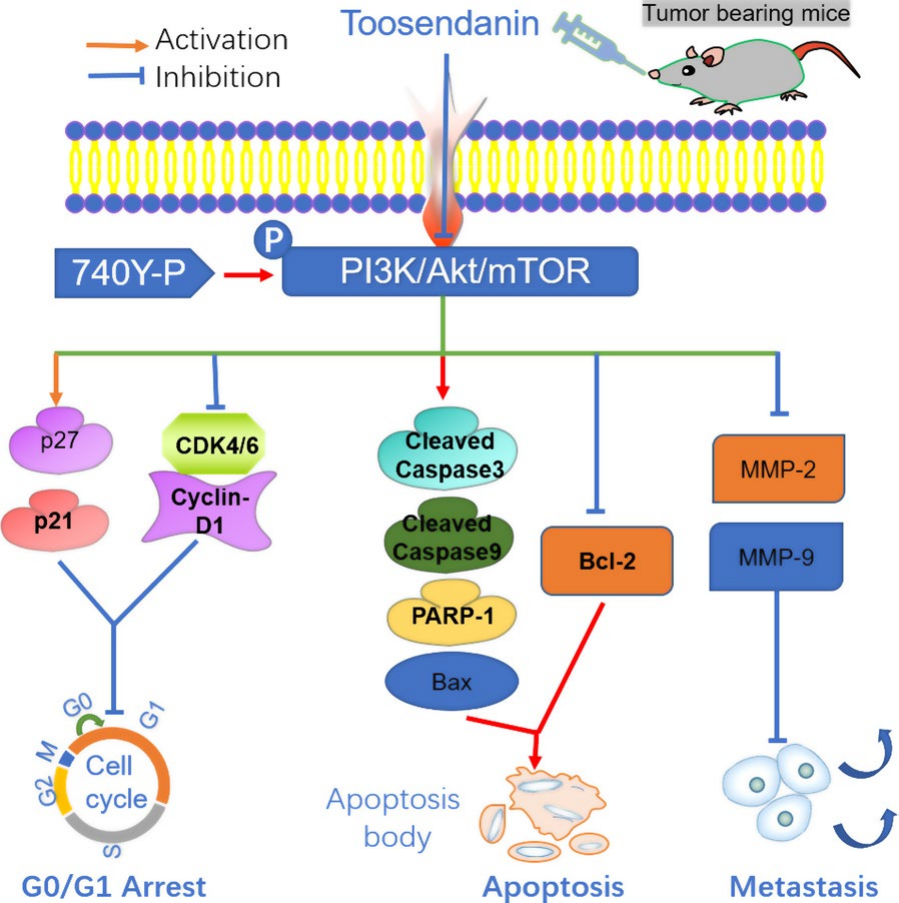
Schematic representation for the potential mechanisms of anti-tumor activity of TSN in glioma cells

This study aimed to evaluate the effect of TSN on the progression of GBM and elucidate potential mechanisms. We found that TSN inhibits tumor progression through the inhibition of PI3K, AkT, and mTOR signaling pathways. Below the figure presents a possible network describing the main mechanisms influenced by TSN (Fig. 7).

GBM is a common primary brain tumor with the highest degree of malignancy. Considering this, a multidisciplinary approach focusing on individualized comprehensive treatment is necessary. The latest large-scale clinical trial showed a 20.9-month overall median survival rate in patients with GBM after surgery, radiotherapy, temozolomide, and tumor treating fields [20]. However, the overall prognosis of patients with GBM remains poor; the current chemotherapy is not ideal due to the drug resistance and adverse effects observed. Therefore, effective and feasible treatments are urgently needed.

Studies have recently shown the anti-tumor effects of TSN on different types of malignant tumors [21]; however, the role of TSN in glioma remains unclear. Studies regarding the anti-glioma role of TSN are limited; however, few have shown that the mechanism involves the p53 and mir-608/notch axis [22, 23]. For the first time, we propose the role of TSN in the inhibition of the viability and metastasis of glioma cells through the promotion of induced apoptosis and inhibition of the PI3K/Akt/mTOR pathway.

First, the CCK-8 assay showed the dose- and time-dependent cytotoxicity of TSN on glioma cells (U87MG, LN18, U251, LN229); furthermore, it showed the limited toxicity of TSN on normal human astrocytes. In addition, we found that the U87MG and LN18 cell lines were more sensitive to TSN; therefore, we selected them as the research objects in the follow-up experiment.

Second, the wound healing, transwell, and western blotting assays confirmed the effect of TSN on migration and invasion. Migration and invasion of malignant tumor cells is a complete and complex process [24]. Studies have shown the key role of MMP in tumor invasion and metastasis through the degradation of protein components in the basement membrane, the main tissue barrier of cell invasion [25–28]. Specifically, high levels of MMP-2/9 expression are observed in the process of glioma development and tumor cell invasion [25, 29]. Therefore, we found that TSN inhibits tumor invasion and migration through decreased MMP-2/9 expression in human glioma cells.

Third, Annexin V-FITC/PI double staining, Hoechst 33342 fluorescent staining, and western blotting assay were used to detect apoptosis. The results showed that TSN effectively induces apoptosis, which involves the rate of apoptosis, changes in apoptotic nuclei, and the expression level of related apoptotic proteins. Apoptosis is known as a form of programmed cell death, and its inhibition leads to unlimited growth of tumor cells [30]. Previous studies have shown that TSN can induce tumor cell apoptosis [15, 23]. These results were consistent with our finding that TSN-induced apoptosis is achieved through the activation of a cascade of reactions including the caspase cascade and increased ratio of Bax/Bcl-2.

In addition, flow cytometry results showed that the TSN treatment group had significantly higher cell ratio in the G0/G1 phase of the cell cycle compared to the control group. P21 and p27 are CDK inhibitors; these bind to cyclins-CDKs complex in the G1 phase, which negatively regulates the cell cycle through the inhibition of the G1/S transition phase of the cycle, which prevents cell proliferation and tumor formation [31, 32]. Additionally, we found that TSN inhibited the expression of CDK-4, CDK-6, and cyclin D1, resulting in the arrest of cells at the G0/G1 phase [33].

Furthermore, recent studies have found a close relation between the activation of the PI3K/Akt/mTOR pathway and the occurrence, development, prognosis, and drug resistance of tumor cells [34, 35]. The Cancer Genome Atlas (TCGA) confirmed that alterations in the PI3K/Akt/mTOR gene were present in 88% of patients with GBM [36]. Additionally, the PI3K/Akt/mTOR signaling pathway is directly involved in the regulation of glioma cell proliferation, apoptosis, and metastasis [37–40]. Therefore, this pathway is a widely used target in the research and development of anti-glioma drugs. TSN significantly inhibited the phosphorylation of PI3K, Akt, and mTOR proteins in glioma cells; furthermore, changes in the levels of expression of MMP proteins, cell-cycle-related proteins, and apoptotic proteins were observed [41]. However, we found that the PI3K activator, 740Y-P, reversed these changes. Inhibition of this pathway can downregulate the expression of MMP-2, cyclin D1, and Bcl-2, thus inhibiting cell metastasis and proliferation and inducing cell apoptosis [41–43]. Therefore, we believe that TSN exerts anti-glioma effects through the PI3K/Akt/mTOR signaling pathway.

To further confirm the anti-glioma cancer effect of TSN in vivo, we established a xenograft tumor model of nude mice by transplanting U87MG cells. The results confirmed the inhibitory effect of TSN on the transplanted tumor with limited side effects on the major organs of the nude mice. Likewise, western blotting with immunohistochemical results showed that TSN had a significant effect on cell proliferation, migration, invasion, apoptosis, cell cycle arrest, and PI3K/Akt/mTOR signaling pathway in vivo.

## Conclusions

Our current research showed that TSN plays a crucial role in the anti-tumor effect on glioma cells in vivo and in vitro through interference of the proliferation, invasion, migration, apoptosis, and cycle distribution, mediated through the inhibition of the PI3K/Akt/mTOR signaling pathway. Collectively, these findings provide evidence that TSN is a potential therapeutic agent for glioma.

## Data Availability

The datasets generated and/or analysed during the current study are included in this published article.

## Abbreviations

GBM: Glioblastoma
DMSO: Dimethyl sulfoxide
DMEM: Dulbecco’s modified Eagle’s medium
FBS: Fetal bovine serum
PBS: Phosphate-buffered saline
CCK-8: Cell counting Kit-8
ECL: Electrochemiluminescence
TBS-T: Tris-buffered saline with Tween-20
MMP2: Matrix metalloproteinase-2
PI3K: Phosphoinositol 3-kinase
Akt: Protein kinase B
p-Akt: Phosphorylated Akt
p-PI3K: Phosphorylated PI3K
mTOR: Mammalian target of rapamycin
p-mTOR: Phosphorylated mTOR
MMP9: Matrix metalloproteinase-9
ATCC: American Type Culture Collection
OD: Optical density
PI: Propidium iodide
SPF: Specific pathogen-free
SD: Standard deviation
IC50: The half inhibitory concentration
